# Establishment of a porcine bronchial epithelial cell line and its application to study innate immunity in the respiratory epithelium

**DOI:** 10.3389/fimmu.2023.1117102

**Published:** 2023-07-03

**Authors:** Kohtaro Fukuyama, Tao Zhuang, Eita Toyoshi, Fernanda Raya Tonetti, Sudeb Saha, Binghui Zhou, Wakako Ikeda-Ohtsubo, Keita Nishiyama, Hisashi Aso, Julio Villena, Haruki Kitazawa

**Affiliations:** ^1^ Food and Feed Immunology Group, Laboratory of Animal Food Function, Graduate School of Agricultural Science, Tohoku University, Sendai, Japan; ^2^ Livestock Immunology Unit, International Education and Research Center for Food and Agricultural Immunology (CFAI), Graduate School of Agricultural Science, Tohoku University, Sendai, Japan; ^3^ Laboratory of Animal Health Science, Graduate School of Agricultural Science, Tohoku University, Sendai, Japan; ^4^ Laboratory of Immunobiotechnology, Reference Centre for Lactobacilli (CERELA-CONICET), Tucuman, Argentina; ^5^ Department of Dairy Science, Sylhet Agricultural University, Sylhet, Bangladesh

**Keywords:** respiratory epithelium, epithelial cells, TLR3, viral immunity, TLR4, porcine respiratory epithelial cell line

## Abstract

*In vitro* culture models that precisely mirror the porcine respiratory epithelium are needed to gain insight into how pathogens and host interact. In this study, a new porcine bronchial epithelial cell line, designated as PBE cells, was established from the respiratory tract of a neonatal pig. PBE cells assumed a cobblestone-epithelial like morphology with close contacts between the cells when they reached confluence. The PBE cell line was characterized in terms of its expression of pattern recognition receptors (PRRs) and its ability to respond to the activation of the Toll-like receptor 3 (TLR3) and TLR4 signaling pathways, which are key PRRs involved in the defense of the respiratory epithelium against pathogens. PBE cells stimulated with poly(I:C) were able to up-regulate the expression of *IFN-β*, *IFN-λ1* (*IL-29*), *IFN-λ3* (*IL-28B*), the antiviral factors *Mx1*, *OAS1*, and *PKR*, as well as the viral PRRs *RIG-1* and *MDA5.* The expression kinetics studies of immune factors in PBE cells allow us to speculate that this cell line can be a useful *in vitro* tool to investigate treatments that help to potentiate antiviral immunity in the respiratory epithelium of the porcine host. In addition, poly(I:C) and LPS treatments increased the expression of the inflammatory cytokines *TNF-α, IL-6, IL-8*, and *MCP-1/CCL2* and differentially modulated the expression of negative regulators of the TLR signaling pathways. Then, PBE cells may also allow the evaluation of treatments that can regulate TLR3- and TLR4-mediated inflammatory injury in the porcine airway, thereby protecting the host against harmful overresponses.

## Introduction

Respiratory tract infections (RTIs) have been a major cause of morbidity and mortality in humans and animals historically. The improved sanitation, the greater access to health care, the research and development in antimicrobials, and the implementation of vaccines have helped to significantly diminish the incidence and severity of RTIs in humans (1). However, these advances have not been paralleled in the prevention of RTIs in animals of economic importance. The large-scale intensified animal production systems with high-density of individuals in confined spaces and the use of genetically homogenous populations in farms have been major drivers of pathogen spread (1). The prevention of RTIs in livestock is of great importance, not only because of the economic impact associated with the loss of animals, weight loss and reduced weight gain (2), but also because of its direct effect on human health. The modern systems for animal production often serve as the interface between wild and human populations, and multiple viral spillover events have occurred at this nexus (1). In addition, animal trade has contributed to multiple outbreaks globally, particularly RTIs. Perhaps the best example is the swine influenza virus (SIV) that has become endemic in pigs worldwide and was able to cross species barrier to infect humans causing the influenza pandemic in 2009 (3).

Pigs are relevant as livestock and RTIs are one of the main causes of economic losses in the swine industry (2). Both, bacterial and viral pathogens can colonize and infect the porcine respiratory tract causing from mild symptoms to severe lung diseases. Furthermore, it was reported that bacterial and viral pathogens can be detected in various combinations in porcine RTIs, indicating that these infectious diseases are often polymicrobial (2, 4). It was suggested that the onset of porcine RTIs is generally related to a primary viral infection that produce alterations in the respiratory mucosa promoting the secondary bacterial colonization (2, 4), as it has been described for humans (5, 6). Primary viral pathogens such as porcine reproductive and respiratory syndrome virus (PRRSV), porcine respiratory coronavirus (PRCV) and SIV are endemic in pig farms (2), and in addition to being a cause of morbidity, they can favor secondary bacterial infections through the damage of the respiratory epithelium and the impairment of mucosal immunity. The efficient prevention of respiratory viral infections in the porcine host could not only help to mitigate their consequences but also to reduce the incidence and severity of secondary bacterial infections.

Pathogens infecting the porcine respiratory mucosa via aerosols and/or droplets often initiate their replication in respiratory epithelial cells (RECs) in the upper tract (7). After this first replication, pathogens can disseminate and infect RECs from the lower respiratory tract inducing more severe diseases such as pneumonia or bronchitis. As seen in other mucosal surfaces, the RECs are at the interface with the environment and therefore they are of key importance in host defense. RECs facilitate mucociliary clearance and produce antimicrobial compounds to avoid the colonization of infectious pathogens (7). In addition, RECs express pattern recognition receptors (PRRs) that recognize structural components of microbes designated as microbial-associated molecular patterns (MAMPs). The recognition of MAMPs by RECs generates immunological changes in these cells that contribute to limit infections and help to coordinate the response of immune cells. In fact, complex interactions exist between RECs and mucosal immune cells that modulate their response to pathogens in a bidirectional way (7). Considering the important role of RECs in the defense of the respiratory mucosa, *in vitro* systems based on these cells have been proposed to advance in the knowledge of pathogen-host interaction as well as to investigate preventive and therapeutic alternatives that help to mitigate the impact of RTIs.

Primary RECs cultures and cell lines of human origins have been successfully used to investigate host-microbe interaction (reviewed in (8). These *in vitro* tools, which accurately represents the host biology because of the expression of relevant host factors and have less ethical concern than animal models, are of value to explore respiratory infections and characterize potential therapeutics for human RTIs (8). On the other hand, RECs of porcine origin have been studied only to a limited extent. In fact, porcine RECs cultures have been used mainly to study the replication, cytopathic effects, and immune responses of influenza virus (IFV) (9–12). Species-specific differences in the response of cells to viral challenges were observed. The Nipah virus (NiV) infects the respiratory tract of both humans and pigs. However, while NiV-infected pigs develop an acute and often severe inflammatory-mediated respiratory disease, symptoms are seen only in few NiV-infected human patients (13). Comparative studies using primary cultures of human and porcine bronchial epithelial cells infected with NiV revealed that both RECs responded to NiV infection by producing IFN-λ and antiviral factors (*OAS* and *ISG-56*). Human cells were more efficient than porcine cells to up-regulate *IFN-λ* and antiviral factors, which correlated with lower viral RNA content (13). Of note, while porcine bronchial epithelial cells had a reduced capacity to produce IFN-λ, they were capable to strongly express the proinflammatory cytokines *IL-6* and *IL-8*, which were suggested to contribute to inflammatory-pathology. Similar results were described for IFV. Human RECs express significantly higher levels of *IFN-β*, *IFN-λ*, *ISG15*, *Mx1*, and *OAS1* after the challenge with IFV than porcine cells. Furthermore, porcine RECs not only mounted an innate immune response that was lower in magnitude, but it was also delayed compared to human cells (14). Altogether, these results highlight the differences in the immune response of human and porcine RECs, indicating that species-specific cells are needed to investigate treatments that have true therapeutic value *in vivo*. Thus, reproducible *in vitro* culture models that precisely mirror the porcine respiratory epithelium are needed to gain insight into how pathogens and host interact. Investigating the expression of PRRs in porcine RECs, the activation and regulation of the related signaling pathways, their detailed immune response to PAMPs, and the influence of respiratory commensal bacteria in such responses are examples of the knowledge that should be generated to better design strategies to protect the porcine host from RTIs.

In the present study, we established a new porcine bronchial epithelial cell line designated as PBE cells from the respiratory tract of a neonatal pig. We characterized the PBE cell line in terms of its expression of PRRs and its ability to respond to the activation of the TLR3 and TLR4 signaling pathways, which are key PRRs involved in the defense of the respiratory epithelium against viral and bacterial pathogens, respectively.

## Materials and methods

### Animals and experimental tissues

Bronchial tissues were obtained from a neonatal LWD pig (Hiruzu Co., Ltd., Miyagi, Japan). The piglet for the bronchial tissue sampling was clinically healthy and free of infectious diseases as assessed by examination of a veterinarian. The piglet was slaughtered by electroshock and bloodletting according to the approved procedures. All procedures were conducted in accordance with the Guidelines for Animal Experimentation of Tohoku University, Japan, under the protocol number 2019 Noudou-038-02.

### Isolation and cloning of PBE cells

Tissue pieces of the bronchus were collected from a 7-day piglet. The epithelial layer of the bronchus was scraped with a razor blade, and then transferred to 15 ml tubes containing serum free Dulbecco’s modified Eagle medium (DMEM, GIBCO, NY, USA), supplemented with penicillin (10 U/ml) and streptomycin (10 µg/ml). The tube was centrifuged at 1,500 rpm for 10 min at 4°C, and then the supernatant was removed. After washing three times with DMEM, the tissue pieces were transferred to collagen coated flasks (Sumilon, Tokyo, Japan) containing 10% FBS-DMEM, and then incubated at 37°C with 5% CO_2_.

In order to establish the PBE cell line, the epithelial cells of the primary cultures were cloned using the limiting dilution method after several passages of the primary culture. The cells were treated with a sucrose/EDTA buffer (pH 7.5; 0.45 M sucrose, 0.36% EDTA in PBS) for 3 min at 37°C, detached using 0.04% trypsin/PBS (GIBCO, NY, USA), and then diluted to 50 cells/ml in 10% FBS-DMEM. The cells were seeded on a collagen-coated 96-well plate (Sumilon, Tokyo, Japan). Each well was checked for cell growth and monoclonal expansion at day 4 after plating by microscopic analysis. A single colony of rapidly growing cells with epithelial-like morphology was found. Then the cells were passaged for the immortalization and the immunocytochemical analysis.

### Immortalization

The porcine bronchial epithelial cells were used for transfection with pSV3-neo (ATCC, MA, USA). This plasmid codes for the oncogene SV40 large T antigen and a neomycin (G418)-resistance gene. For transfection, 1×10^6^ cells were electroporated and treated with 1µg of SV40 large t antigen DNA per tip in 10 µl in the Neon™ Transfection System (Invitrogen, CA, USA) with. The cells were seeded in a collagen-coated 6 well plate containing 10% FBS-DMEM with 200 µg/ml G418 for the selection of transfected cells. After 5 days culture, the cells were collected and transferred to flasks for further characterization and maintained in culture.

### Immunocytochemical staining

The PBE cells were seeded on a collagen-coated 8-well culture-slide at a cell density of 2×10^4^ cells/cm^2^ for several days, washed with cold PBS once and then fixed with methanol and acetone (vol/vol) for 15 min at -20°C. After washing three times with PBS, the cells were treated with bovine serum albumin containing PBS for 20 min at room temperature. After washing three times with PBS, the cells were incubated with mouse monoclonal anti-SV40 T-antigen antibody (1/50 dilution, Abcam, CA, UK) or mouse monoclonal anti-cytokeratin, pan (mixture) antibody (1/50 dilution, Sigma-Aldrich, MA, USA). The cells were also incubated with rabbit polyclonal anti-ZO-1 antibody (1/2000 dilution, 21773-1-AP, Proteintech, IL, USA), mouse monoclonal anti-occludin antibody (1/3000 dilution, 66378-1-lg, Proteintech, IL, USA), rabbit anti-alpha-tubulin antibody (1/100 dilution, Proteintech, IL, USA), rabbit anti-E-cadherin antibody (1/200 dilution, Proteintech, IL, USA), rabbit anti-muc5B antibody (1/100 dilution, Proteintech, IL, USA), rabbit polyclonal anti-TLR3 antibody (1/100 dilution, GTX113022, Genetex, CA, USA), rabbit polyclonal anti-TLR4 antibody (1/100 dilution, BS-1021R, Bioss Antibodies, MA, USA) or rabbit polyclonal anti-TLR7 antibody (1/100 dilution, BS-6601R, Bioss Antibodies, MA, USA) overnight at 4°C in the dark. The PBE cells were washed three times with PBS, treated with secondary Alexa Fluor 488 conjugated Goat anti-rabbit IgG antibody (1/500 dilution, A-11008, Thermo Fisher Scientific, MA, USA), or Alexa Fluor 488 conjugated Goat anti-mouse IgG antibody (1/500 dilution, A-11001, Thermo Fisher Scientific, MA, USA) for 1h at 4°C in the dark. The PBE cells were washed three times with PBS, treated with secondary Alexa Fluor 488 conjugated donkey anti mouse IgG antibody (1/200 dilution, Jackson Immuno Research, PA, USA), or Alexa Fluor 488 conjugated donkey anti rabbit IgG antibody (1/200 dilution, Jackson Immuno Research, PA, USA) for 1h at -4°C in the dark. After three times washing by PBS, the cells were counterstained with 40,6-diamidino-2-phenylindole (DAPI) for 5 min at room temperature in the dark, and then washed three times with PBS. Cells treated only with secondary antibodies but not with the primary antibodies were used as controls. Slide images were viewed using a Laser Scanning Microscope BZ-9000 (Keyence, Tokyo, Japan), and photographed at 200× with software BZ II Viewer, Version 1.4.0.0.

### Cell viability

PBE cells were seeded on a collagen-coated 24 well plate (MS0024, Sumitomo Bakelite, Tokyo, Japan) at an initial concentration of 0.5×104 cells/cm^2^ or 1×10^4^ cells/cm^2^. Cells were collected by Accutase (12679-54, NACALAI TESQUE, Kyoto, Japan) after washing with PBS and mixed cell suspension and Trypan blue stain 1:1. Cell counts were determined by a blood cell counting board every day for 6 days.

### Transepithelial electrical resistance analysis

PBE cells were seeded on a collagen-coated inserts 24-well plate (0.4 μm pore size, 354444, Corning, AZ, USA) at an initial concentration of 1×10^5^, 1.5×10^5^ or 2.0×10^5^ cells/well. Transepithelial electrical resistance (TEER) was measured every 2 days of cultivation of PBE cells in transwell inserts using an epithelial volt–ohm meter with a chopstick electrode (Millicell ERS-2, MERS00002, EMD Millipore, Billerica, MA). Triplicate measurements were recorded for each monolayer.

### Quantitative expression analysis by real-time PCR

PBE cells were seeded at an initial concentration of 1.0 x 10^4^ cells/cm^2^. At day 6, PBE cells were stimulated with the TLR3 synthetic agonist poly(I:C) (100 ng/ml) or the TLR4 agonist LPS (1000 ng/ml). The expressions of immune factors were evaluated at several points after the treatments by quantitative real time PCR. The total RNA of PBE cells was isolated by using the TRIzol reagent (Invitrogen, Carlsbad, CA, USA) according to the manufacturer’s instructions. The concentration and purity of isolated RNA was determined with NanoDrop^®^ ND-1000 Spectrophotometer. Reverse transcription was performed with the PrimeScript RT reagent Kit (Takara Bio, Shiga, Japan) following the manufactures instructions. The quantitative real time PCR was conducted on a CFX Connect Real-time PCR System (Bio-rad, Hercules, CA, USA) using TB Green Premix Ex Taq (Takara Bio, Shiga, Japan) according to the manufacturer’s recommendations. The thermal cycling conditions were 95°C for 30 followed by 40 cycles at 95°C for 5 s and 60°C for 30 s. The primers used were listed in **Supplementary Table 1**. The β-actin, which is stably expressed in various tissues of pigs, was used as a housekeeping gene (15, 16). The expression level of mRNA was calculated using the calibration curve obtained from serially diluted plasmids, which was normalized by the expression level of β-actin in each sample, and then expressed as relative with the control set as 1.

### Statistical analysis

Experiments were performed in triplicate and results were expressed as mean ± standard deviation (SD). After verification of the normal distribution of data, 2-way ANOVA was used. Tukey’s test (for pairwise comparisons of the means) was used to test for differences between the groups. Differences were considered significant at p<0.05.

## Results

### Establishment of PBE cells

The PBE cell line was developed using the simian virus 40 large T antigen (SV40). Primary culture cells were transfected with SV40 to create an immortalized cell line (**Supplementary Figure 1**). Although primary cells only survive between 3-4 passages, the immortalized PBE cells were successfully passaged more than 20 times.

**Figure 1:**
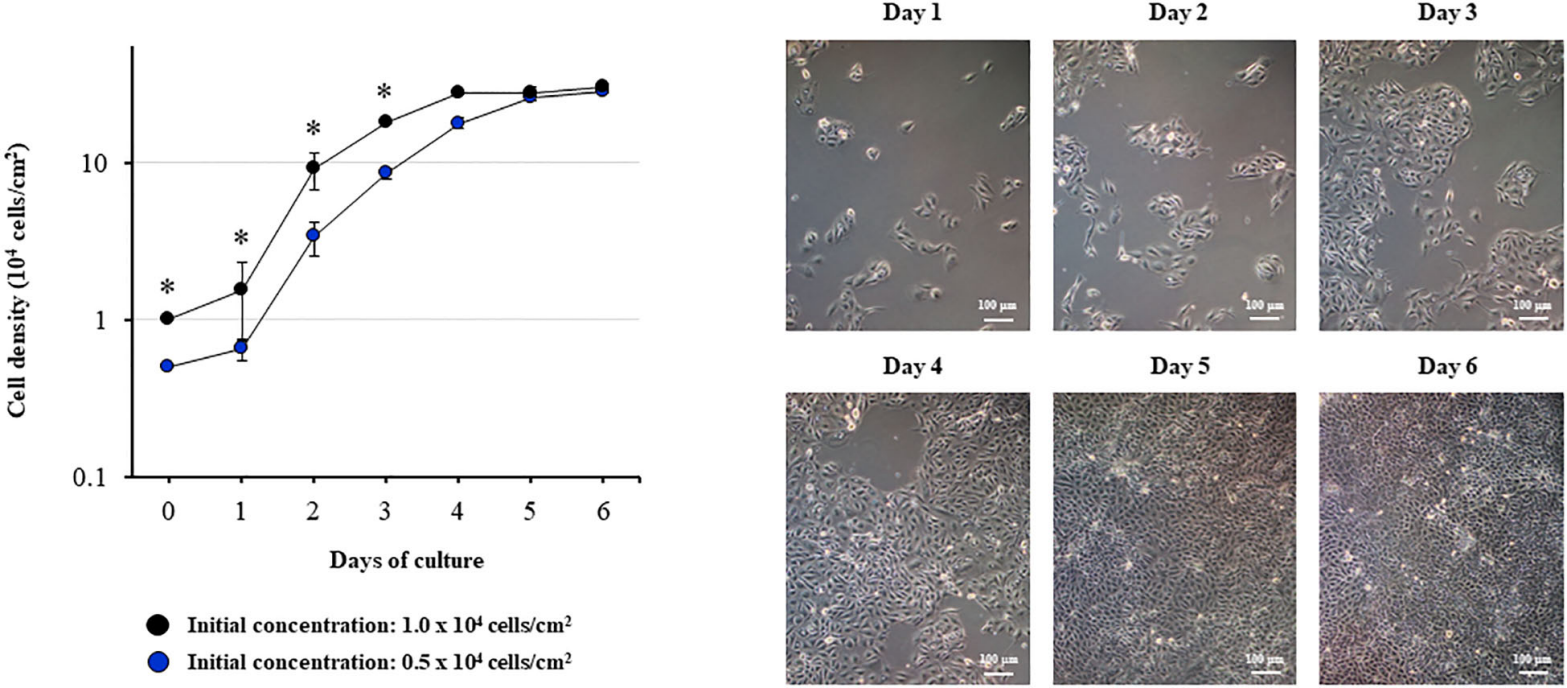
Growth kinetics of the originally established porcine bronchial epithelial (PBE) cell line. PBE cells were seeded at an initial concentration of 0.5 x 10^4^ or 1.0 x 10^4^ cells/cm^2^ and evaluated during six days to determine cell density. Microscope photographs show the growth of cells seeded at an initial concentration of 0.5 x 10^4^ cells/cm^2^ from day 1 to day 6, in which the cells assumed a cobblestone-epithelial like morphology with close contacts between the cells. Results represent data from three independent experiments. Asterisks indicate significant differences in cell densities between the curves with distinct initial concentrations. * (P < 0.05).

In order to evaluate the growth kinetics of the PBE cells, two initial concentrations (0.5 x 10^4^ or 1.0 x 10^4^ cells/cm^2^) were used, and cells were evaluated until they reached confluence (**Figure 1**). The cells grew continuously until day 5, reaching confluence between days 5 and 6. The different initial concentration was reflected in different cell densities up to day 4. However, no significant differences in cell densities were observed between days 5 and 6 for the different initial concentrations. Microscopic analysis demonstrated that the PBE cells assumed a cobblestone-epithelial like morphology with close contacts between the cells when they reached confluence (**Figure 1**). The initial seeding concentration of 1.0 x 10^4^ cells/cm^2^ was selected for further experiments.

**Figure 2:**
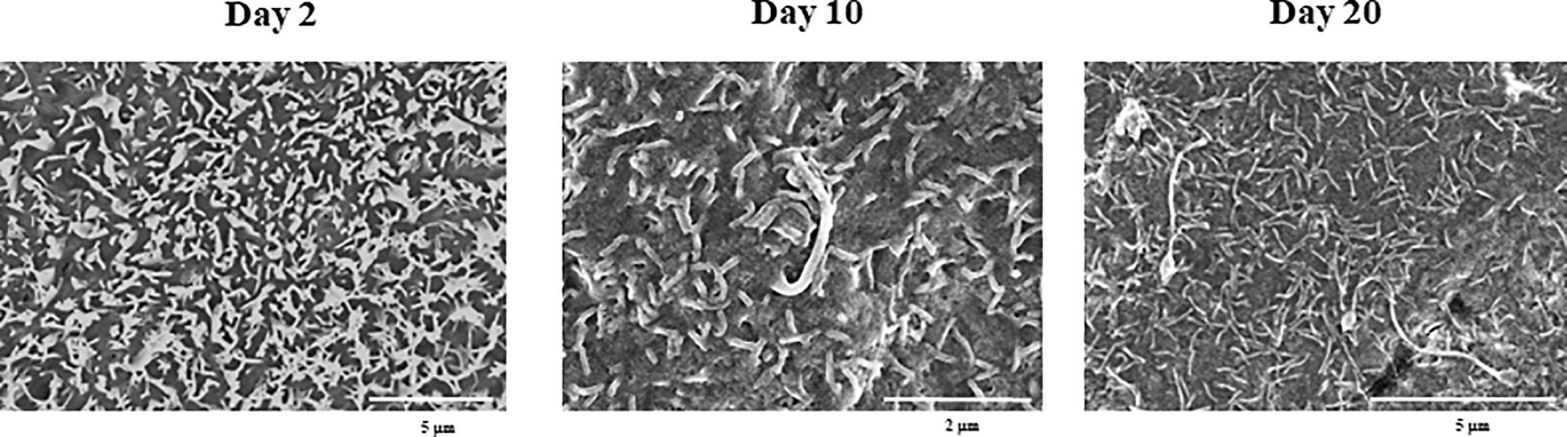
Scanning electron microscopy (SEM) characterization of the originally established porcine bronchial epithelial (PBE) cell line. PBE cells were seeded at an initial concentration of 1.0 x 10^4^ cells/cm^2^ and evaluated at days 2, 10 and 20 by SEM analysis. SEM photographs show the surface of PBE cells in which the formation of cilia that increase in size over time can be distinguished. Results represent data from two independent experiments.

**Figure 3:**
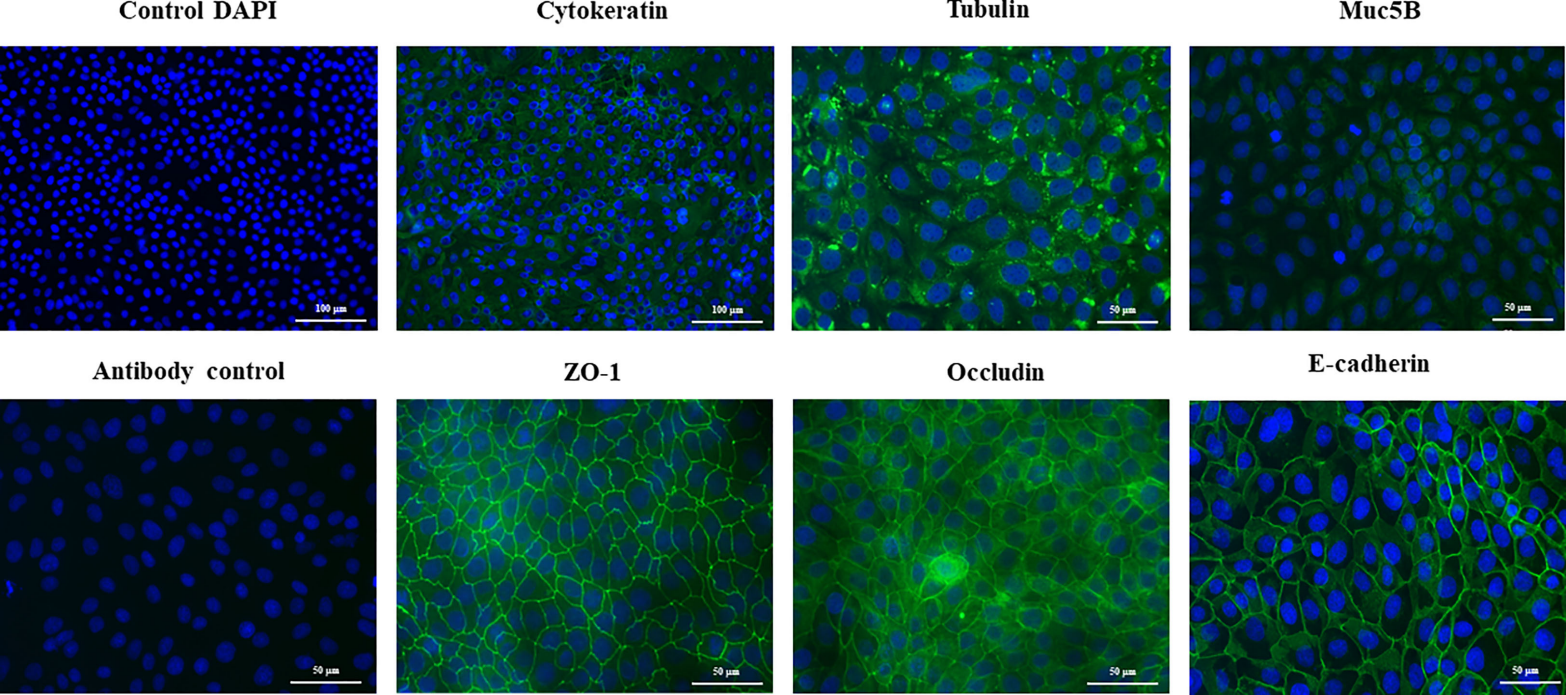
Immunohistochemical characterization of the originally established porcine bronchial epithelial (PBE) cell line. PBE cells were seeded at an initial concentration of 1.0 x 10^4^ cells/cm^2^ and evaluated at day 6. Photographs show the PBE cells stained with fluorescent antibodies directed to cytokeratin, tubulin, muc5B protein, the peripheral membrane phosphoprotein zona occludens 1 (ZO-1), occludin or E-cadherin. Cell nuclei were stained with DAPI. Antibody controls were performed by incubating cells with secondary antibodies without the addition of primary antibodies. The photographs show antibody control for ZO-1. Results represent data from two independent experiments.

SEM microscopic analysis was performed to characterize the surface of PBE cells at days 2, 10 and 20 (**Figure 2**). SEM study revealed the formation of cilia on the apical surface of PBE cells, which increased in size over time. This feature is a characteristic of respiratory epithelial cells. To further evaluate the epithelial nature of PBE cells, we performed immunohistochemical analysis to determine the expression of cytokeratin. PBE cells uniformly expressed cytokeratin as shown in **Figure 3**, confirming that PBE cells possess an epithelial phenotype. Tubulin was also evaluated in PBE cells and results demonstrated a strong expression of this protein (**Figure 3**). In addition, we studied the expression of proteins involved in cell-to-cell contact in the PBE cell line. It is well known that tight junctions or zona occludens (ZO) allow close contact between epithelial cells contributing to the maintenance of cell polarity and blocking the movement of transmembrane proteins between the apical and the basolateral cell surfaces. The ZO-1 function as an adaptor protein that link junctional transmembrane proteins, such as occludin and claudin, to the actin cytoskeleton. Thus, we studied the expression of ZO-1, occludin and E-cadherin in PBE cells (**Figure 3**). The expression of the three proteins was clearly observed in the PBE cells, particularly in the cell-to-cell contact regions. To measure the integrity of tight junction dynamics in PBE cells cultures the TEER analysis was applied. It was observed that the electrical resistance of the cellular monolayer gradually increased during a 11-days period (**Supplementary Figure 2**), confirming the establishment of a barrier. A low production of mucus was detected in PBE cells (data not shown). In order to confirm the production of mucus by the new cell line, we evaluated the expression of muc5B protein (**Figure 3**). The immunofluorescence study demonstrated a low expression of muc5B in the PBE cells.

Our results indicate that PBE cells reach a 100% confluent monolayer by 6 days of culture. Then, confluent PBE cells that have separated the apical and basolateral compartments were used for further experiments.

### Expression of PRRs in PBE cells

**Figure 4:**
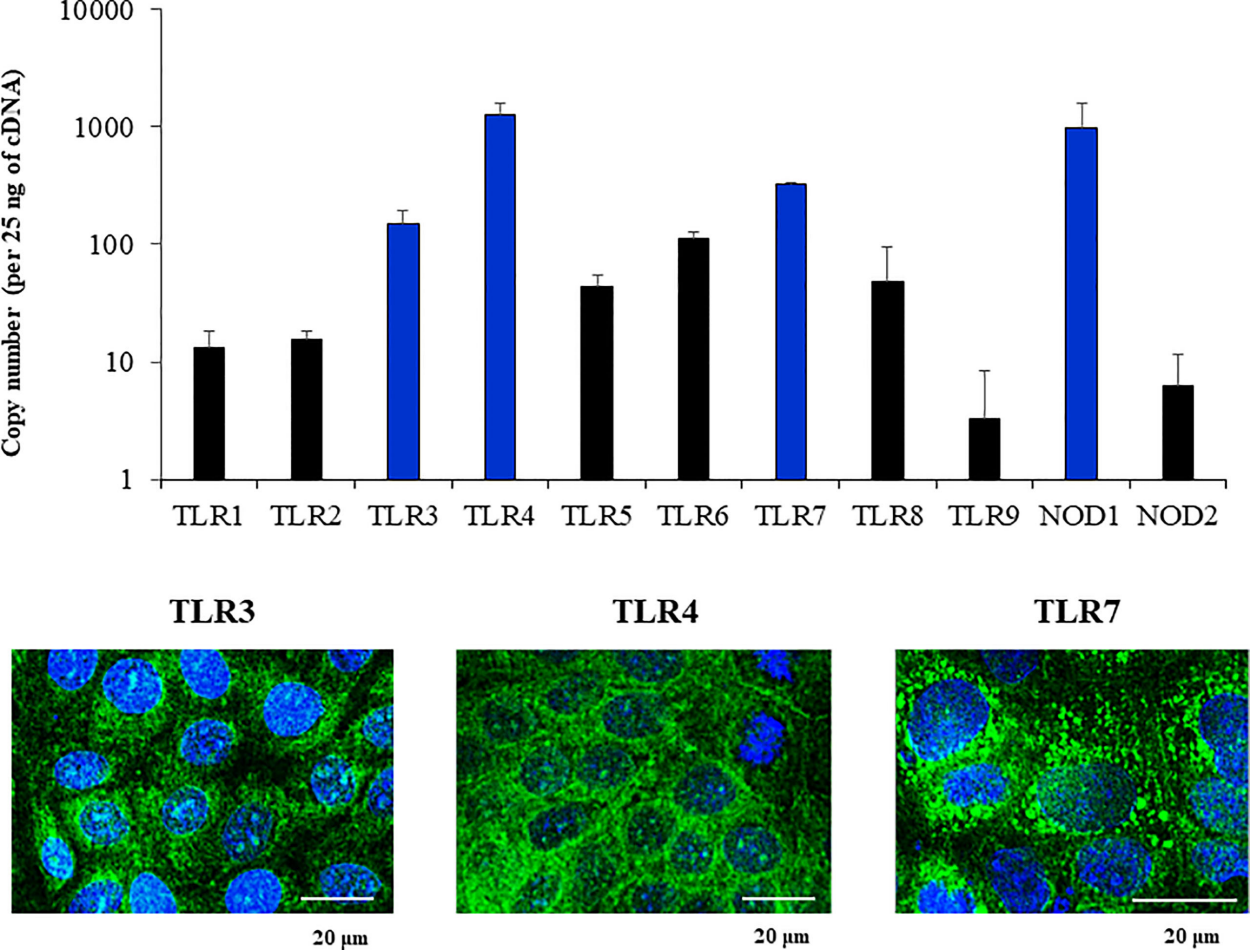
Expression of Pattern Recognition Receptors (PRRs) in the originally established porcine bronchial epithelial (PBE) cell line. PBE cells were seeded at an initial concentration of 1.0 x 10^4^ cells/cm^2^ and evaluated at day 6. The expressions of PRRs from the Toll-like receptor (TLR) and the nucleotide-binding oligomerization domain-containing protein (NOD) families were evaluated by qPCR. Results are expressed as copy numbers of the PRRS genes per 25 ng of cDNA. Photographs show the PBE cells stained with fluorescent antibodies directed to TLR3, TLR4, or TLR7. Cell nuclei were stained with DAPI. Results represent data from three independent experiments.

We next aimed to evaluate whether the PBE cell line expressed relevant genes of the PRRs families that are involved in the recognition of microorganisms in the respiratory epithelium. We focused our studies in the expressions of PRRs from the Toll-like receptor (TLR) and the nucleotide-binding oligomerization domain-containing protein (NOD) families. The qPCR analysis revealed that PBE cells express *TLR1-9*, being *TLR3*, *TLR4* and *TLR7* the ones more highly expressed in this cell line (**Figure 4**). We also detected the expression of *NOD1* and *NOD2* in PBE cells (**Figure 4**). In order to confirm the expression of TLR3, TLR4 and TLR7 and to evaluate their cellular location, we performed immunohistochemical analysis (**Figure 4**). Both TLR3 and TLR7 were detected in a granular pattern in areas close to the cell nucleus, indicating their expression in endosomes. As expected, TLR4 was detected near the nucleus of cells but also in the cellular membrane (**Figure 4**).

### Response of PBE cells to the activation of TLR3 signaling pathway

**Figure 5:**
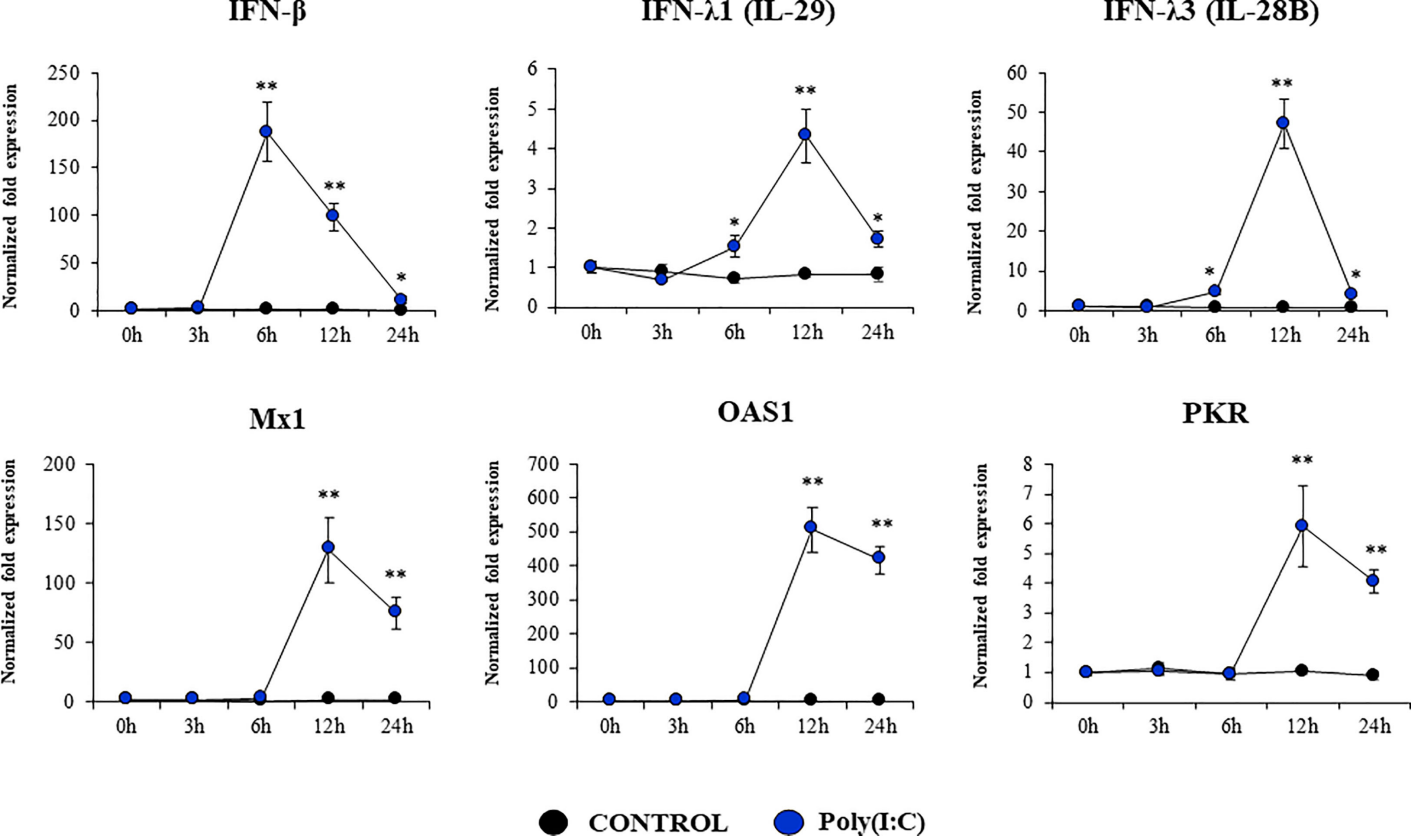
Expression of interferons (IFNs) and antiviral factors in the originally established porcine bronchial epithelial (PBE) cell line in response to the activation of the Toll-like receptor 3 (TLR3) signaling pathway. PBE cells were seeded at an initial concentration of 1.0 x 10^4^ cells/cm^2^. At day 6, PBE cells were stimulated with the TLR3 synthetic agonist poly(I:C) (100 ng/ml) and the expressions of *IFN-β*, *IFN-λ1* (*IL-29*), *IFN-λ3* (*IL-28B*) and the antiviral factors *Mx1*, *OAS1*, and *PKR* were evaluated by qPCR at the indicated time points. Results represent data from three independent experiments. Asterisks indicate significant differences between the control and the poly(I:C)-treated PBE cells. * (P < 0.05), ** (P < 0.01).

**Figure 6:**
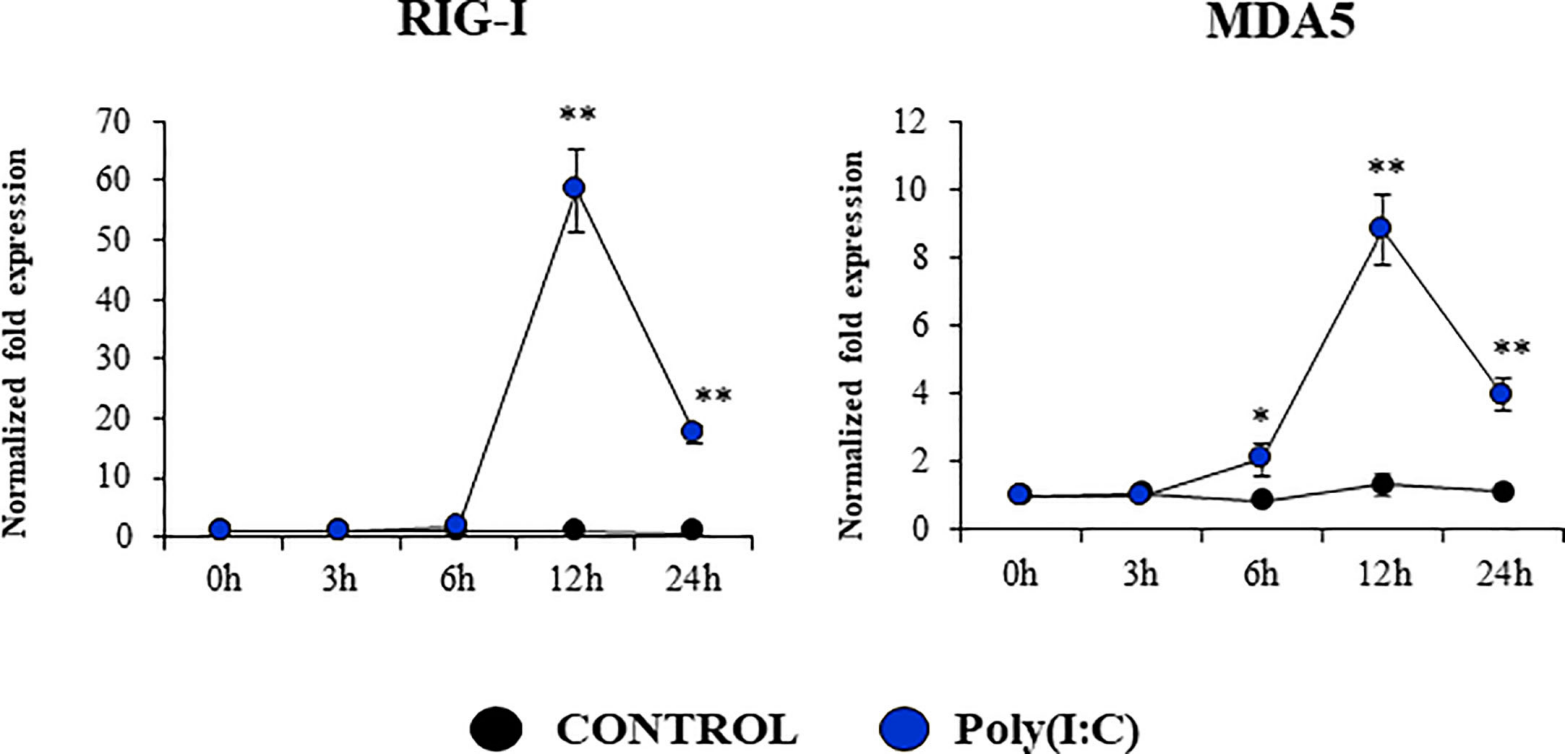
Expression of antiviral Pattern Recognition Receptors (PRRs) in the originally established porcine bronchial epithelial (PBE) cell line in response to the activation of the Toll-like receptor 3 (TLR3) signaling pathway. PBE cells were seeded at an initial concentration of 1.0 x 10^4^ cells/cm^2^. At day 6, PBE cells were stimulated with the TLR3 synthetic agonist poly(I:C) (100 ng/ml) and the expressions of the PRRs *RIG-1*, and *MDA5* were evaluated by qPCR at the indicated time points. Results represent data from three independent experiments. Asterisks indicate significant differences between the control and the poly(I:C)-treated PBE cells. * (P < 0.05), ** (P < 0.01).

Considering the strong expression of TLR3 in PBE cells and the relevant role of this PRR in the defense of the respiratory epithelium against viral pathogens, we next aimed to characterize their response to the TLR3 signaling pathway activation. PBE cells were stimulated with the synthetic TLR3 agonist poly(I:C) and the expressions of *IFN-β*, *IFN-λ1* (*IL-29*), *IFN-λ3* (*IL-28B*) and the antiviral factors *Mx1*, *OAS1*, and *PKR* were evaluated by qPCR at several time points (**Figure 5**). Poly(I:C) stimulation increased the expression of *IFN-β*, *IFN-λ1*, and *IFN-λ3* in PBE cells. A peak at hour 6 was observed for *IFN-β*, which then started to decrease until hour 24. For *IFN-λ1* and *IFN-λ3* the peak was detected later at hour 12 (**Figure 5**). Consistent with the peak of *IFN-β* at hour 6, the IFN-induced genes *Mx1*, *OAS1*, and *PKR* were significantly increased from hour 12 and remained elevated until hour 24 (**Figure 5**). The changes in the expression of the viral PRRs *RIG-1* and *MDA5* were also evaluated (**Figure 6**). *MDA5* was significantly increased in PBE cells stimulated with poly(I:C) from hour 6, reaching a peak at hour 12. On the other hand, *RIG-I* was enhanced from hour 12 (**Figure 6**).

**Figure 7:**
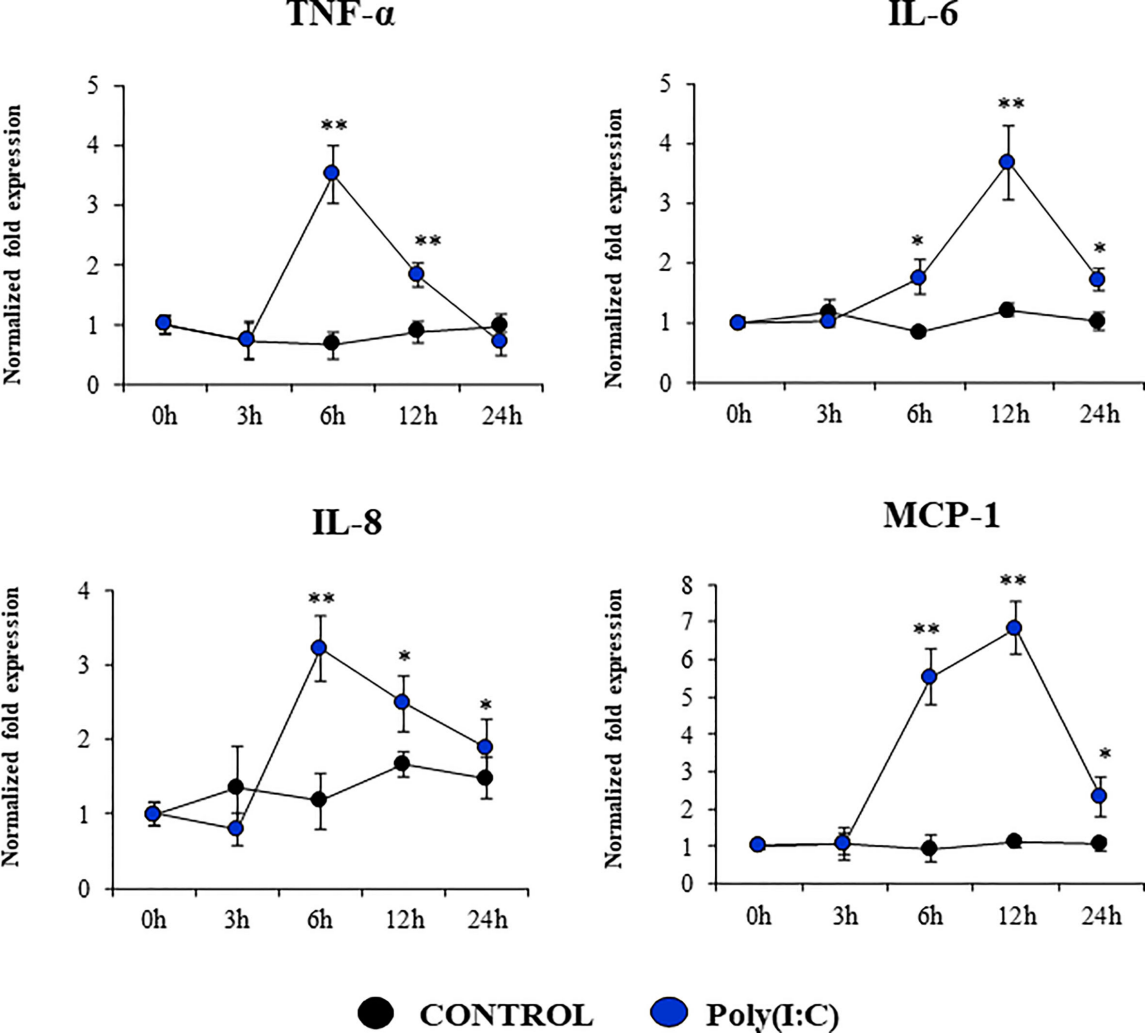
Expression of inflammatory cytokines and chemokines in the originally established porcine bronchial epithelial (PBE) cell line in response to the activation of the Toll-like receptor 3 (TLR3) signaling pathway. PBE cells were seeded at an initial concentration of 1.0 x 10^4^ cells/cm^2^. At day 6, PBE cells were stimulated with the TLR3 synthetic agonist poly(I:C) (100 ng/ml) and the expressions of the inflammatory factors *TNF-α, IL-6, IL-8*, and *MCP-1* were evaluated by qPCR at the indicated time points. Results represent data from three independent experiments. Asterisks indicate significant differences between the control and the poly(I:C)-treated PBE cells. * (P < 0.05), ** (P < 0.01).

We also evaluated the changes in the expressions of inflammatory cytokines and chemokines in PBE cells stimulated with poly(I:C) (**Figure 7**). The activation of the TLR3 signaling pathway in PBE cells triggered the up-regulation of *TNF-α, IL-6, IL-8*, and *MCP-1/CCL2. TNF-α* and *IL-8* showed a peak at hour 6 and then decreased gradually until hour 24. The peak for the expression of *IL-6* and *MCP-1* was observed at hour 12 (**Figure 7**).

**Figure 8:**
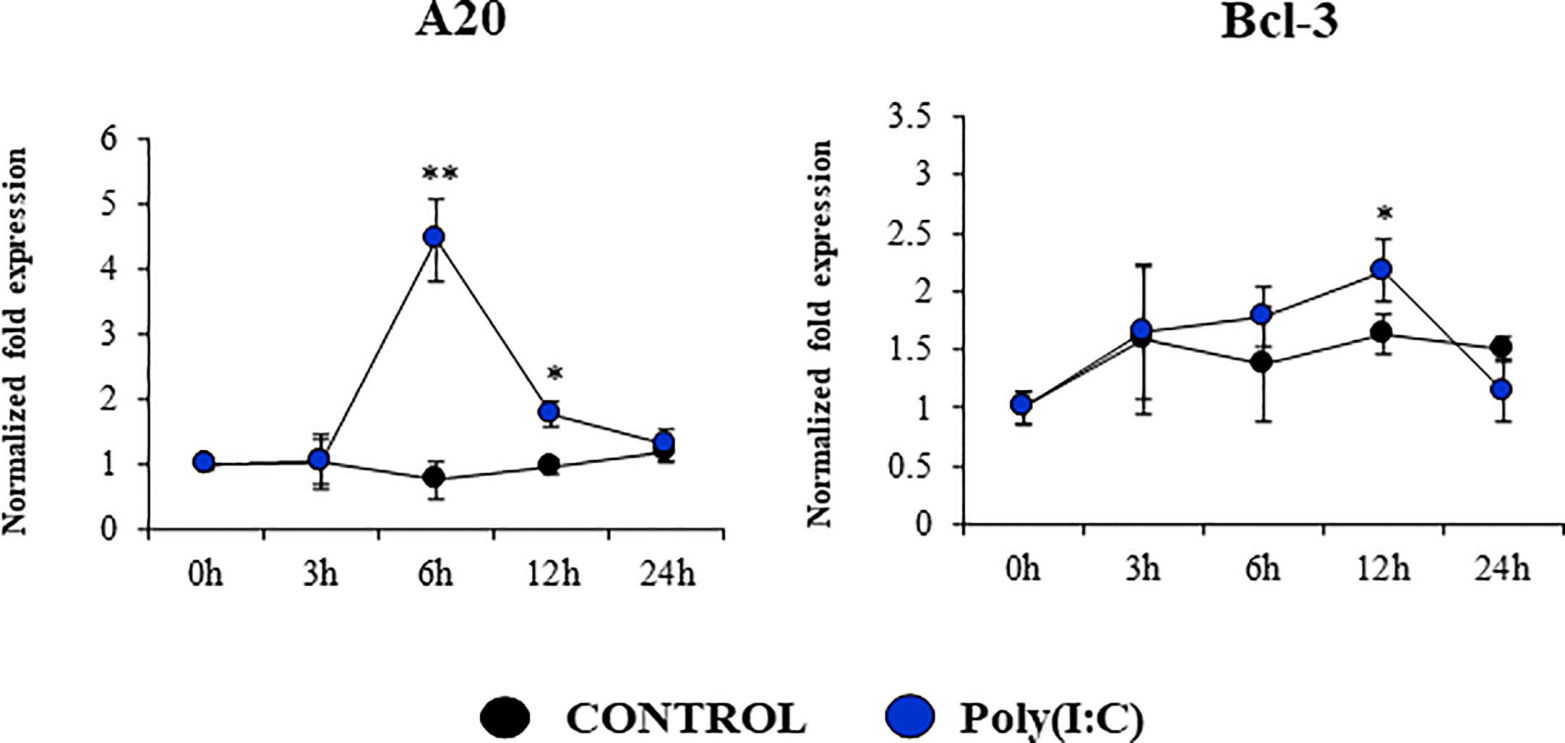
Expression of negative regulators of the Toll-like receptor (TLR) signaling pathway in the originally established porcine bronchial epithelial (PBE) cell line in response to the activation of TLR3. PBE cells were seeded at an initial concentration of 1.0 x 10^4^ cells/cm^2^. At day 6, PBE cells were stimulated with the TLR3 synthetic agonist poly(I:C) (100 ng/ml) and the expressions of the TLR negative regulators *A20* and *Bcl-3* were evaluated by qPCR at the indicated time points. Results represent data from three independent experiments. Asterisks indicate significant differences between the control and the poly(I:C)-treated PBE cells. * (P < 0.05), ** (P < 0.01).

We assessed whether poly(I:C) treatment modified the expression of negative regulators of the TLR signaling pathway in PBE cells (**Figure 8** **,** **Supplementary Figure 3**). The expressions of the TLR negative regulators *A20, Bcl-3, IRAK-M*, *SIGIRR*, *MKP-1*, and *Tollip* were evaluated at several time points after TLR3 activation. No significant changes were detected for the expression of *SIGIRR*, *IRAK-M*, *Tollip* and *MKP-1* when poly(I:C)-stimulated PBE cells were compared to unstimulated controls (**Supplementary Figure 3**). Slight but statistically significant increase of *Bcl-3* were observed at hour 12 (**Figure 8**). The most notable change in the expression of the TLR negative regulators was observed for *A20*. An increase of more than 4-fold expression was detected for *A20* at hour 6, which then decreased until hour 24 (**Figure 8**).

### Response of PBE cells to the activation of TLR4 signaling pathway

**Figure 9:**
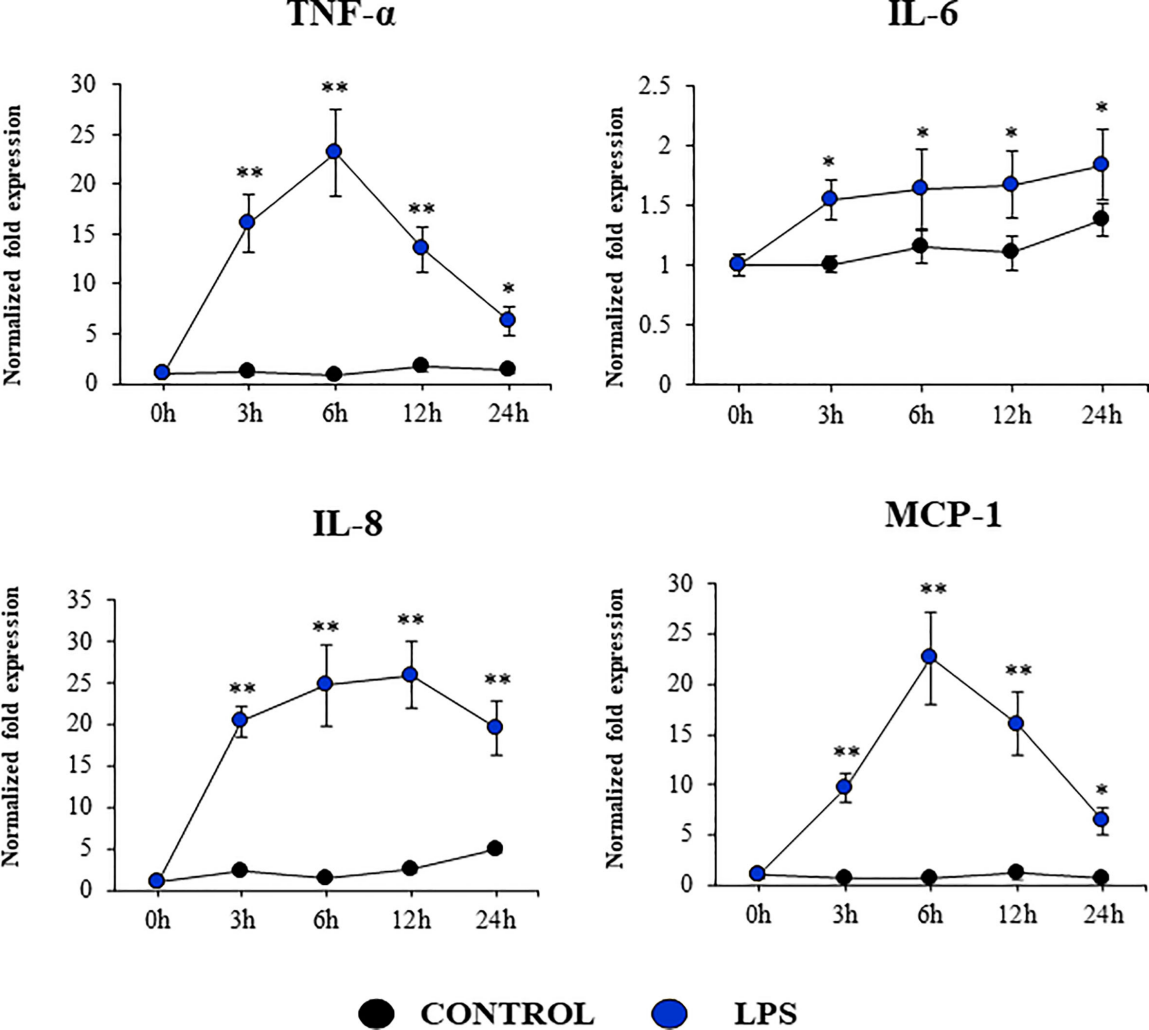
Expression of inflammatory cytokines and chemokines in the originally established porcine bronchial epithelial (PBE) cell line in response to the activation of the Toll-like receptor 4 (TLR4) signaling pathway. PBE cells were seeded at an initial concentration of 1.0 x 10^4^ cells/cm^2^. At day 6, PBE cells were stimulated with the TLR4 agonist LPS (1000 ng/ml) and the expressions of the inflammatory factors *TNF-α, IL-6, IL-8*, and *MCP-1* were evaluated by qPCR at the indicated time points. Results represent data from three independent experiments. Asterisks indicate significant differences between the control and the poly(I:C)-treated PBE cells. * (P < 0.05), ** (P < 0.01).

**Figure 10:**
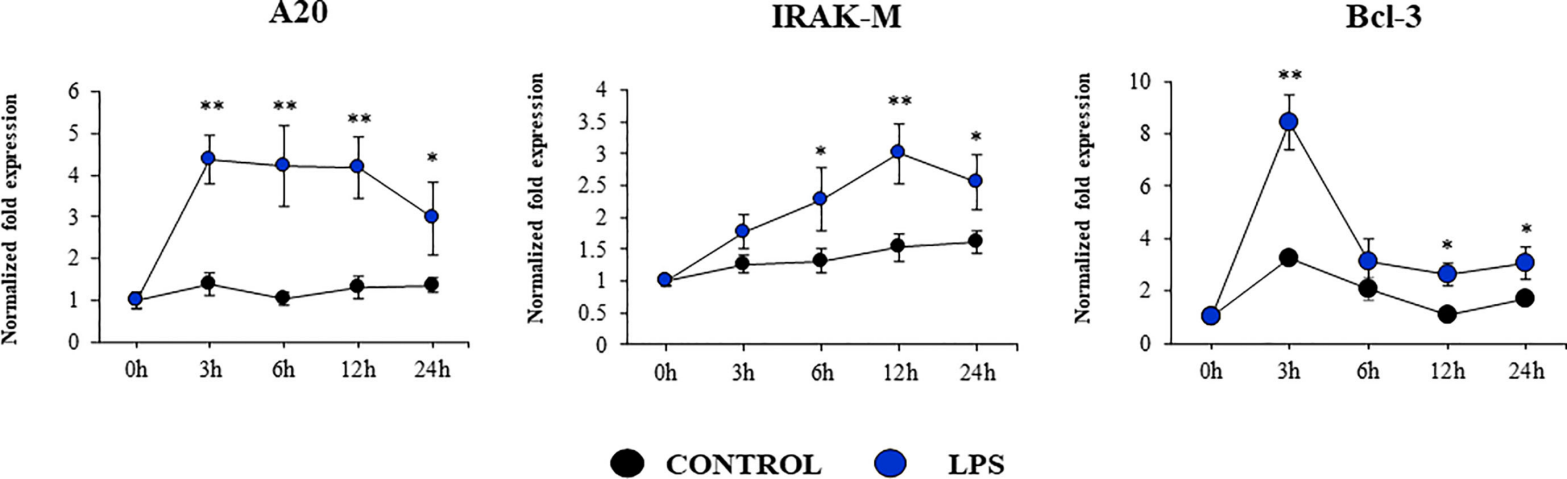
Expression of negative regulators of the Toll-like receptor (TLR) signaling pathway in the originally established porcine bronchial epithelial (PBE) cell line in response to the activation of TLR4. PBE cells were seeded at an initial concentration of 1.0 x 10^4^ cells/cm^2^. At day 6, PBE cells were stimulated with the TLR4 agonist LPS (1000 ng/ml) and the expressions of the TLR negative regulators *A20, IRAK-M* and *Bcl-3* were evaluated by qPCR at the indicated time points. Results represent data from three independent experiments. Asterisks indicate significant differences between the control and the poly(I:C)-treated PBE cells. * (P < 0.05), ** (P < 0.01).

Our studies showed a strong expression of TLR4 in PBE cells (**Figure 4**). Considering the important role of this PRR in the inflammatory response triggered by LPS in the respiratory epithelium, we next aimed to characterize the response of PBE cells to the TLR4 signaling pathway activation. PBE cells were stimulated with the TLR4 agonist LPS and the expressions of *TNF-α, IL-6, IL-8*, and *MCP-1* were evaluated at several time points (**Figure 9**). *TNF-α* and *MCP-1* showed a peak at hour 6 and then decreased gradually until hour 24. The peak for the expression of *IL-8* was observed between hours 6 and 12; while *IL-6* elevated its expression from hour 3 and was maintained at those levels until the end of the studied period (**Figure 9**). When we evaluated the expressions of the TLR negative regulators in PBE cells after TLR4 activation, it was observed that *SIGIRR*, *MKP-1*, and *Tollip* were not modified (**Supplementary Figure 4**). In contrast, LPS stimulation significantly augmented the expression of *A20, Bcl-3*, and *IRAK-M* in PBE cells (**Figure 10**). An increase of *Bcl-3* was observed at hour 3, which then decreased until hour 24 while the expression of *IRAK-M* showed a peak at hour 12 (**Figure 10**). An increase of more than 4-fold expression was detected for *A20* between hours 3 and 12, which then decreased at hour 24 (**Figure 10**).

## Discussion

The RECs lining the airway mucosa are key actors in host defense because of their capacity to interact with cells of the immune system (7, 17). Epithelial cells of the respiratory mucosa recognize pathogens via PRRs leading to the production of IFNs, cytokines and chemokines that render them and their neighboring cells in an alert state and cooperate to recruit and activate immune cells (18). Then, besides a barrier role, RECs have inherent innate immunity functions and for that reason they are of great interest in the study of immunity to infections. Both, primary RECs cultures and cell lines of human and porcine origins have been successfully used to investigate host-microbe interactions.

The use of primary RECs cultures has some limitations. The comparison of studies using primary cultures of RECs is not easy, because of the differences in the anatomic source of the cells, the use of undifferentiated versus differentiated cells, the culture methods as well as the donor variability (reviewed in (8). In addition, it was shown that primary RECs cultures are often contaminated with fibroblasts and can be passaged a limited number of times before the loss of epithelial integrity and senescence occurs (10), which is frequently accompanied by an altered gene expression (19). These facts significantly limit the number of experiments that can be performed with primary cultures. To avoid these disadvantages, models based on immortalized cell lines were developed, which were proved to be useful in the study of infections and immune responses at a cellular and molecular levels (8). Although there are several cell lines of human origin, there are less examples of porcine RECs lines (10, 20). A porcine lung epithelial cell line designated St. Jude porcine lung cells (SJPL) was established and proposed as a useful *in vitro* tool to investigate IFV replication (21) and immunity (22). However, it was demonstrated later that the SJPL cell line is not of porcine origin but of monkey origin (23). A porcine cell line designated as newborn pig trachea (NPTr) was developed by serial culture of primary cells (20). The work demonstrated that several types of porcine virus were capable to replicate in this cell line although no detailed studies of the immune responses were performed. Immortalized porcine bronchial epithelial cells (PBECs) were established by Xie et al. (24), by transfecting primary cells with human telomerase reverse transcriptase. Authors demonstrated that immortalized PBECs retained the morphological and functional features of primary RECs as indicated by proliferation and cytokeratin expression assays. The work also reported that this cell line is susceptible to SIV and porcine circovirus (24). Porcine nasal and tracheal respiratory epithelial cells were also immortalized to develop the siNEC and siTEC cell lines, respectively. Both cell lines were capable to form tight junctions and cilia as well as to support IFV replication (10). Similarly, the porcine airway cell line (MK1-OSU) derived from the distal trachea of a 5-week-old piglet has been useful for the evaluation of IFV infection (12). Then, porcine RECs lines have been used to a limited extent and mainly to research in IFV infection. Here, we developed a PBE cell line from the bronchial epithelium of a neonatal pig and demonstrated that these cells are able to grow in laboratory conditions, reach confluence and express tight junction proteins and cilia. Furthermore, we showed that PBE cells express PRRs and respond to both TLR3 and TLR4 signaling pathways activation.

Tight junctions and adherent junctions help to selectively regulate the paracellular diffusion of molecules and form a barrier against invading pathogens in the respiratory epithelium (25). This characteristic is of key importance in REC lines that should be preserved despite immortalization. It was shown that porcine respiratory cell lines like siTEC cells retained the abilities to form tight junctions and to form cilia (10). Similarly, we observed that the new PBE cell line reached confluence, expressed ZO-1, occludin and E-cadherin, and developed cilia. In addition, it was demonstrated that bronchial epithelial cells express functional TLR1-6 and TLR9 (26). Thus, RECs are equipped with PRRs, such as TLRs and NODs, which rapidly sense pathogens and initiate immune responses in the respiratory tract (27). The expression of functional PRRs is also an important characteristic of cell lines aimed to evaluate immunity in the respiratory epithelium. In this regard, it was shown that MK1-OSU cells expressed TLRs-2, -4, -9, RIG-I, and MDA5 (12). TLR-3 was not detected in this cell line and authors proposed that this fact was related to the lack of cross-reactivity of human detection antibodies with swine antigens. The expression of TLR3 mRNA levels was not investigated. In this work, we demonstrated that PBE cells express TLR1-9, NOD1 and NOD2. Furthermore, we showed that TLR3 is one of the most strongly expressed PRRs and we characterized the response of PBE cells to the activation of this signaling pathway by up-regulating antiviral factors as well as inflammatory cytokines and chemokines.

Type I and III IFN induction is a potent mechanism of protection against viral infections. It was reported that primary porcine bronchial epithelial cells responded to synthetic dsRNA stimulation upregulating the expression of *IFN-β* and *IFN-λ* (14). Furthermore, porcine RECs pretreated with poly(I:C) and then challenged with IFV had viral titers that were significantly lower than cells infected only with IFV (14). In line with these results, primary cultures of RECs from the trachea and bronchus of pigs have been shown to significantly up-regulate the expression of the antiviral factors *Mx1* and *ISG15* in response to both poly(I:C) and IFV challenges (11). The work also demonstrated that the inhibition of the JAK/STAT signaling pathway significantly increased the IFV replication. Furthermore, the challenge of porcine RECs with the avian IFV H1N1/06 induced a reduced expression of antiviral factors allowing the virus to replicate efficiently and to cause detrimental effects on cells. Interestingly, the pretreatment of porcine RECs with poly(I:C) diminished IFV replication via the paracrine IFN-β stimulation (11). The expression kinetics studies of *IFN-β*, *IFN-λ1*, *IFN-λ3* and antiviral factors in PBE cells allow us to speculate that this cell line can be a useful *in vitro* tool to investigate treatments that help to potentiate antiviral immunity. In this regard, using a porcine intestinal epithelial cell line developed by our group, we demonstrated that beneficial microorganisms with immunomodulatory capacities differentially regulate genes involved in antiviral defenses enhancing the ability of epithelial cells limit rotavirus replication (28–30). PBE cells could be used for the *in vitro* selection and characterization of beneficial microorganisms with the ability to regulate TLR3-signaling pathway in the respiratory tract allowing an improved epithelial innate antiviral immune response. Research on this topic is ongoing in our laboratories.

We also detected the up-regulation of *RIG-I* and *MDA5* in PBE cells stimulated with poly(I:C). RIG-I and MDA5 are present in the cytoplasm and detect viral nucleic acids leading to the production of IFNs, cytokines and chemokines (31, 32). RIG-I stimulates IFNs production during IFV infection (14). It was shown that the stimulation of the respiratory cell lines NSBE or NHBE with dsRNA enhance the expression of RIG-I, which contributes to increased amplification of IFN responses (14). It was also demonstrated that IFV infection increase the expressions of MDA5 in both *in vivo* and *in vitro* studies (32, 33). Furthermore, the mRNA levels of *MDA5* were significantly increased after the challenge of MK1-OSU cells with SIV (34, 35). These results further highlight the potential of PBE cells for conducting detailed antiviral immunological studies. One limitation of our study is the lack of use of a real viral challenge such as IFV to evaluate the response of PBE cells. Studying the ability of PBE cells to allow the replication of respiratory viruses of importance for the porcine host as well as evaluating the immune responses that are triggered by viral infections are important topics for future research.

The production of inflammatory factors in PBE cells was also characterized considering that the respiratory epithelium is sensitive to the inflammatory damage. It was reported that TNF-α can damage the integrity of the epithelial barrier using a porcine tracheal epithelial cell model. TNF-α can disrupt both ZO-1 and occludin and induce the production of IL-6 and IL-8 (36). It was also shown that the activation of TLR3 pathway in the respiratory tract can have both protective and detrimental effects, the later associated with inflammatory-mediated damage. The nasal administration of poly(I:C) to mice induce the production of pro-inflammatory mediators and the recruitment of inflammatory cells in the respiratory tract, which mediate tissue damage and impairment of lung function (37, 38). *In vitro* studies reported that the treatment of RECs with poly(I:C) stimulate the secretion of multiple inflammatory cytokines and chemokines including TNF-α, IL-6, IL-8, and MCP-1 (39, 40). Similarly, the stimulation of RECs with LPS can trigger the activation of the innate immune response characterized by an improved production of inflammatory factors. It was shown that human alveolar epithelial cells (A549) and bronchial epithelial cells (NHBE) activate the NF-κB inducing the production of the inflammatory cytokines TNF-α, IL-6, and IL-8 (41, 42). In line with these previous studies, we showed here that the stimulation of PBE cells with poly(I:C) or LPS significantly augmented the expression of *TNF-α, IL-6, IL-8*, and *MCP-1.*

Interestingly, we also detected variations in the expression of negative regulators of the TLR signaling pathway in PBE cell stimulated with TLR3 or TLR4 agonists, particularly in *A20* (encoded by *Tnfaip3*) and *Bcl-3*. Research have demonstrated that TLR negative regulators may have a relevant role in the development of inflammatory diseases of the respiratory tract. The chronic exposure to low-doses of LPS protected mice from developing house dust mite-induced asthma. LPS treatment diminished the production of cytokines by RECs that activate dendritic cells and potentiate type 2 immunity. Of note, *A20* knockdown in lung epithelium abolished the protective effect of LPS (43). The chronic and exaggerated inflammation in the airways in cystic fibrosis has been also associated to the reduced expression of A20 in the respiratory epithelium (43). Moreover, A20 protein production is induced in the lung from mice and human bronchial epithelial cells upon IFV infection and its overexpression in bronchial epithelial cells results in the protection of host against inflammatory damage (44). Human NCI-H292 airway epithelial cells stimulated with LPS significantly increase their production of IL-8 as well as the level of *Tnfaip3* mRNA (45). Furthermore, overexpression of *A20* inhibited activation of both NF-kB and the IL-8 promoter. Similarly, tracheobronchial epithelial cells (TBEC) showed a strong production of IL-8 and IP-10 (CXCL10) in response to poly(I:C) treatment, which was accompanied with the upregulation of *A20* and *IRAK-M* (39). As the A20 protein, IRAK-M is involved in immunoregulation of airway inflammation. Although the role of IRAK-M in the respiratory tract immune responses was demonstrated by its influence on macrophages, dendritic cells, and T cells, recent studies highlighted its role in RECs (46). It was demonstrated that the knockdown of IRAK-M in EAS-2B and A549 cells significantly increased their ability to produce IL-6, IL-8, CXCL10, and CXCL11 in response to IL-1β, TNF-α, or IL-33 stimulation. On the other hand, it was shown that the high upregulation of *IL-8* expression induced by respiratory syncytial virus (RSV) infection in A549 cells is terminated by Bcl-3 in the late-phase of infection. In contrast to wild-type mice, Bcl-3-deficient mice exhibited significantly increased susceptibility toward the Gram-negative pathogen *Klebsiella pneumoniae* (47, 48). The loss of Bcl-3 generated a remarkable cytokine imbalance in the lungs, which was characterized by increased production of the neutrophil-attracting chemokines. Taken together, our results suggest that the expression of inflammatory cytokines and chemokines as well as TLR negative regulators in PBE cells is a valuable characteristic of this new cell line that may allow the evaluation of treatments that can regulate TLR-mediated inflammatory injury in the porcine airway.

The applications of the new porcine cell line developed in this work could be extended beyond its use in monolayers, for example it could be used in co-cultures with immune cells. Single cell RNA-seq and computational analysis (7) have started to reveal the complex epithelial–immune crosstalk that occurs in the respiratory tract in health and disease. Bi-directional interactions between RECs and alveolar macrophages help to maintain the homeostatic state of tolerance to innocuous antigens and the appropriate protective responses to pathogens when required (49). Most of these studies have been done in mouse models (50) while few studies investigated epithelial–macrophage crosstalk in humans (51). To the best of our knowledge, no studies have been performed in the porcine host. This new PBE cell line could be used with porcine alveolar macrophages (52) to evaluate their interactions in the context of the response to pathogens or PRRs activation.

A limitation of our work is that it was not possible to compare the immune responses mediated by TLR3 or TLR4 activation of PBE cells with those produced under the same conditions by primary cultures of porcine bronchial epithelial cells. Carrying out these studies would be of great value to propose this new cell line as a highly representative model of the immunological responses that occur in the bronchial epithelium *in vivo*. Furthermore, it would be of great interest to carry out these comparative studies with primary cultures of bronchial epithelial cells from pigs of different ages to assess whether the PBE cells represent only a limited age group. These are studies that we intend to do in the immediate future.

In conclusion, the PBE cell line developed in this work was characterized in terms of its expression of PRRs and its ability to respond to the activation of the TLR3 and TLR4 signaling pathways, which are key PRRs involved in the defense of the respiratory epithelium against pathogens. PBE cells stimulated with poly(I:C) were able to up-regulate the expression of *IFN-β*, *IFN-λ1* (*IL-29*), *IFN-λ3* (*IL-28B*), the antiviral factors *Mx1*, *OAS1*, and *PKR*, as well as the viral PRRs *RIG-1* and *MDA5.* In addition, poly(I:C) and LPS treatments increased the expression of the inflammatory cytokines *TNF-α, IL-6, IL-8*, and *MCP-1/CCL2* and differentially modulated the expression of negative regulators of the TLR signaling pathways. The expression kinetics studies of immune factors in PBE cells allow us to speculate that this cell line can be a useful *in vitro* tool to investigate treatments that help to potentiate antiviral immunity and/or regulate TLR-mediated inflammatory injury in the porcine airway, thereby protecting the host against harmful overresponses.

## Data availability statement

The raw data supporting the conclusions of this article will be made available by the authors, without undue reservation.

## Ethics statement

The animal study was reviewed and approved by Animal Experimentation of Tohoku University.

## Author contributions

JV and HK designed the study and wrote the manuscript. KF, TZ, FR, ET, SS, and BZ did the laboratory work. KF, TZ, and FR performed statistical analysis. KF, WI-O, KN, HA, JV, and HK contributed to data analysis and interpretation. All authors read and approved the manuscript.
