# Supplementary figures and images for: Establishment of a porcine bronchial epithelial cell line and its application to study innate immunity in the respiratory epithelium
Source: Front Immunol. 2023 Jul 3;14:1117102. doi: 10.3389/fimmu.2023.1117102 (PMC10350646; doi:10.3389/fimmu.2023.1117102)

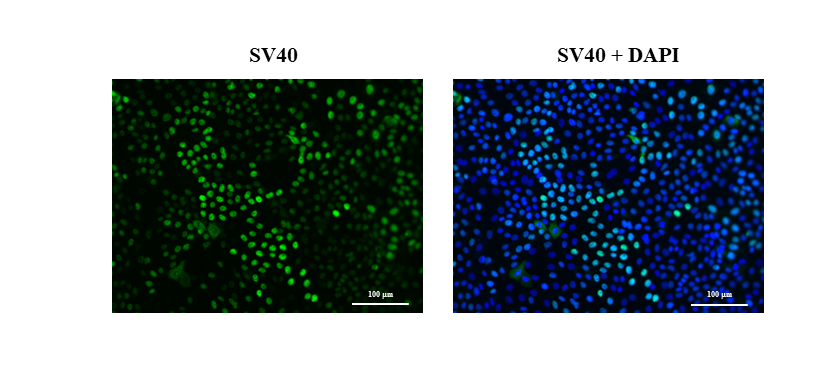

Supplement: Supplementary file 1 [file Image_1.tif]

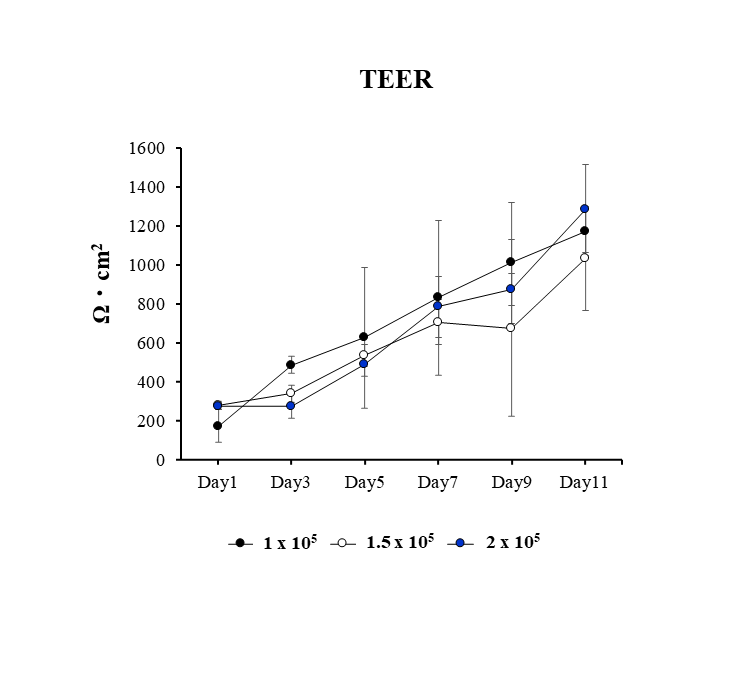

Supplement: Supplementary file 2 [file Image_2.tif]

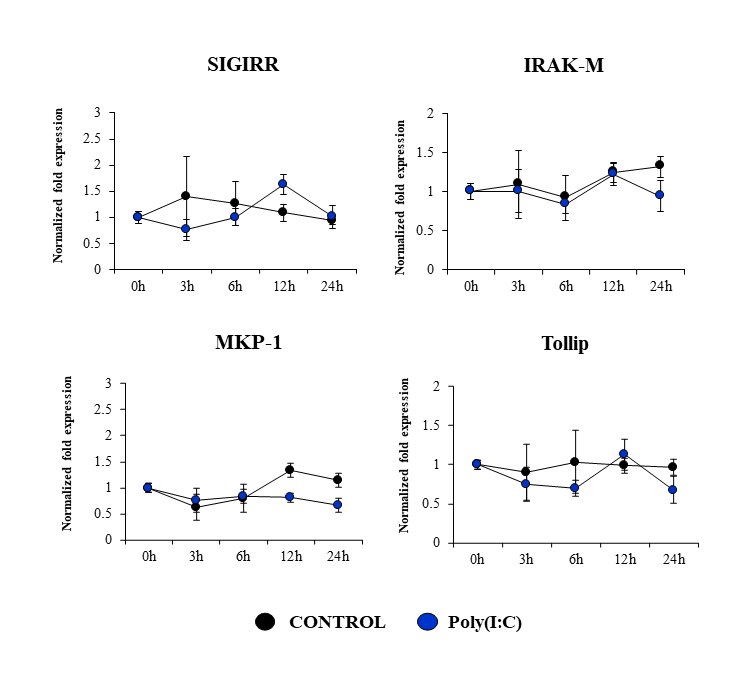

Supplement: Supplementary file 3 [file Image_3.tif]

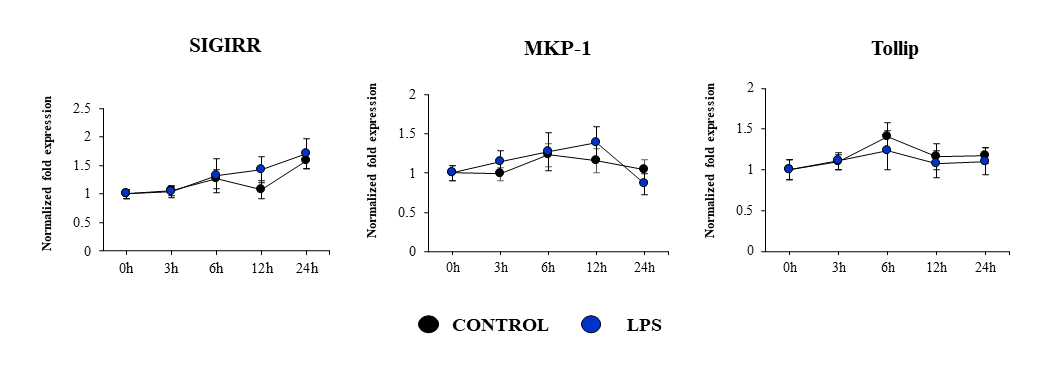

Supplement: Supplementary file 4 [file Image_4.tif]
