# Supplementary material for: Establishment of a porcine bronchial epithelial cell line and its application to study innate immunity in the respiratory epithelium
Source: Front Immunol. 2023 Jul 3;14:1117102. doi: 10.3389/fimmu.2023.1117102 (PMC10350646; doi:10.3389/fimmu.2023.1117102)
Supplement: Supplementary file 5 [file Table_1.docx]

| **NO.** | **Target** | **Forward** | **Reverse** |
| --- | --- | --- | --- |
| 1 | β-actin | CAT CAC CAT CGG CAA CGA | GCG TAG AGG TCC TTC CTG ATG T |
| 2 | TLR1 | TAA TGC ATT CGA TGC CCT GC | TAT GCC AGA CAA ACT GGA GG |
| 3 | TLR2 | ACA TGA AGA TGA TGT GGG CC | TAG GAG TCC TGC TCA CTG TA |
| 4 | TLR3 | TAG AGA CAT GGA TTG CTC CC | AAC TTC TGG AAT GCA GGT CC |
| 5 | TLR4 | CTC TGC CTT CAC TAC AGA GA | CTG AGT CGT CTC CAG AAG AT |
| 6 | TLR5 | TTT CTG GCA ATG GCT GGA CA | TGG AGG TTG TCA AGT CCA TG |
| 7 | TLR6 | TCC CAG GAT CAA GGT TCT TG | GAG CAG AGT CCC CTT ATA AC |
| 8 | TLR7 | GAG TGG GTT TTG GAT GAG CT | CTC TGG GAA AGG TTT TCC AG |
| 9 | TLR8 | CGC CTCTGG GAA TTT TTT GG | AAG AGG TTT CTC AGA GGC TG |
| 10 | TLR9 | GTG GAA CTG TTT TGG CAT C | CAC AGC ACT CTG AGC TTT GT |
| 11 | NOD1 | CTG TCG TCA ACA CCG ATC CA | CCA GTT GGT GAC GCA GCT T |
| 12 | NOD2 | GAG CGC ATC CTC TTA ACT TTC G | ACG CTC GTG ATC CGT GAA C |
| 13 | IFN-β | AGT TGC CTG GGA CTC CTC AA | CCT CAG GGA CCT CAA AGT TCA T |
| 14 | IFN-λ1 | CCT TAG AGG CTG AGC TAG ACT TGA C | AGC CTG AAG TTC GAC GTG GAT G |
| 15 | IFN-λ3 | GTT CAA GTC TCT GTC CCC ACA AG | CTC CAA GAG GGA CTC TTC AAA GG |
| 16 | Mx1 | GAG GTG GAC CCC GAA GGA | CAC CAG ATC CGG CTT CGT |
| 17 | OAS1 | GAG CTG CAG CGA GAC TTC CT | TGC TTG ACA AGG CGG ATG A |
| 18 | PKR | CCC TGC ACT TCT AGC CAT CT | CGAC CAC TGG CCA TTT CTT TC |
| 19 | RIG-I | TAT CCG AGC AGC AGG CTT TG | CTC GTT GCT GGG ATC TAT GGA A |
| 20 | MDA-5 | GCT ACG TGA ACC CCG ATC TC | AAG CTT GTC CAC CAC TGT AGG |
| 21 | TNF-α | TGGCCCAAGGACTCAGATCAT | ACTCTGCCATTGGAGCTGTC |
| 22 | IL-6 | TGG ATA AGC TGC AGT CAC AG | ATT ATC CGA ATG GCC CTC AG |
| 23 | IL-8 | GCT CTC TGT GAG GCT GCA GTT | TTT ATG CAC TGG CAT CGA AGT T |
| 24 | MCP-1 | ACA GAA GAG TCA CCA GCA GCA A | GCC CGC GAT GGT CTT G |
| 25 | A20 | CCT CCC TGG AAA GCC AGA A | GTG CCA CAA GCT TCC TCA CTT |
| 26 | Bcl-3 | CGA CGC GGT GGA CAT TAA G | ACC ATG CTA AGG CTG TTG TTT TC |
| 27 | IRAK-M | TGG AGC AGC CTT GAA TCC TT | TGG ATA ACA CGT TTG GGA ATC TT |
| 28 | SIGIRR | ATG TGA AGT GTC GGC TCA ATG T | TTC ATC TCC ACC TCC CCA TAC T |
| 29 | MKP-1 | TCC GAA TTC CAC TGG GTT CC | ATG TTG GTC CCC AAT GTG CT |
| 30 | Tollip | TAC CGT GGG CCG TCT CA | CCG TAG TTC TTC GCC AAC TTG |

Table 1. Sequence of the primers used in this work.
